# Associations of high altitude polycythemia with polymorphisms in *EPAS1, ITGA6* and *ERBB4* in Chinese Han and Tibetan populations

**DOI:** 10.18632/oncotarget.21420

**Published:** 2017-09-30

**Authors:** Yiduo Zhao, Zhiying Zhang, Lijun Liu, Yao Zhang, Xiaowei Fan, Lifeng Ma, Jing Li, Yuan Zhang, Haijin He, Longli Kang

**Affiliations:** ^1^ Key Laboratory for Molecular Genetic Mechanisms and Intervention Research on High Altitude Disease of Tibet Autonomous Region, Xizang Minzu University, Xianyang 712082, Shaanxi, China; ^2^ Key Laboratory of High Altitude Environment and Gene Related to Disease of Tibet Ministry of Education, School of Medicine, Xizang Minzu University, Xianyang 712082, Shaanxi, China

**Keywords:** high altitude polycythemia, *EPAS1*, *ITGA6*, *ERBB4*, case-control study

## Abstract

High altitude polycythemia (HAPC) is a common chronic disease at high altitude, which is characterized by excessive erythrocytosis (females, hemoglobin ≥ 190 g/L; males, hemoglobin ≥ 210 g/L). It is the most common disease in chronic mountain sickness casued primarily by persistent arterial hypoxia and ventilatory impairment. However, the disease is still unmanageable and related molecular mechanisms remain largely unclear. This study aims to explore the genetic basis of HAPC in the Chinese Han and Tibetan populations. Subjects were screened for HAPC using the latest approved diagnostic criteria. To explore the hereditary basis of HAPC and investigate the association between three genes (*EPAS1, ITGA6, ERBB4*) and HAPC in Chinese Han and Tibetan populations. We enrolled 100 patients (70 Han, 30 Tibetan) with HAPC and 100 healthy control subjects (30 Han, 70 Tibetan). Subjects were screened for HAPC using the latest approved diagnostic criteria combined with excessive erythrocytosis and clinical symptoms. Analysis of variance was used to evaluate the impact of polymorphism on HAPC based on genetic variation. The Chi-squared test and analyses of genetic models, rs75591953 and rs75984373 in *EPAS1*, rs6744873 in *ITGA6*, rs17335043 in *ERBB4* showed associations with reduced HAPC susceptibility in Han populations. Additionally, in Tibetan populations, rs3749148 in *ITGA6*, rs934607 and rs141267844 in *ERBB4* showed a reduced risk of HAPC, whereas rs6710946 in *ERBB4* increased the risk of HAPC. Our study suggest that the polymorphisms in the *EPAS1, ITGA6* and *ERBB4* correlate with susceptibility to HAPC.

## INTRODUCTION

A French doctor noted for the first time that the number of red blood cells (RBCs) increased in the plateau in 1980 [1], this is the first report of HAPC. Hemoglobin concentration increases within a certain range due to hypoxia environment when low-altitude populations migrate to plateau region, and this response is crucial for them to acclimatize the high altitude. Han people who live in the high altitude environment for a long time are prone to chronic mountain sickness, which is characterized by symptoms of long-term hypoxia [2, 3]. On the contrary, most of the Tibetans resided at altitude of 3000 m to 4500 m for a long time possess heritable adaptations to the hypoxic environment [4]. Tibetans have an unique genetic advantage to adapt to hypoxia environment, because they have lower hemoglobin and hematokrit levels. In addition, Tibetans have stronger hypoxia tolerance. These features help them adapt to high altitude and hypoxic conditions. However, a part of Tibetans who showed high level of hemoglobin may also develop into HAPC. Excessive erythrocytosis leads to significant increases in blood viscosity and microcirculation disturbance, which can lead to tissue hypoxia, stroke, myocardial infarction [5, 6]. Thus, altitude polycythemia as reported earlier may actually be indicative of pathological response rather than an adaptive biological process. The prevalence of HAPC among Qinghai-Tibetan Plateau populations was 5% to 18% [7]. In the human groups around the world, native Tibetans are regarded as the one adapted best to living in high altitude areas, and their hemoglobin concentration significantly lower than Han population. It is considered that this characteristic is largely genetic. In addition, the incidence of HAPC in the Tibetan population was significantly lower than Han population, and many evidence suggested that genetic factors contributed to the development of plateau-related diseases.

First, a sequencing of exons scan comparing indigenous highlanders of the Tibetan Plateau with related lowland Han revealed a significant divergence across 30 SNPs located in *EPAS1, ITGA6* and *ERBB4*. In particular, The hypoxia-inducible factor (HIF) 2α encoded by *EPAS1* gene stimulate the production of RBCs, and increasing the concentration of hemoglobin. Expression of *EPAS1* is limited to organs that are involved in oxygen transport and metabolism [8]. Moreover, it was also found that *EPAS1* was associated with high aititude pulmonary [9], which is a special disease when the Han population into the plateau environment. Genetic studies of high altitude in Tibetans have shown that *EPAS1* has been subjected to strong natural selection by the high environment. *EPAS1* non-coding DNA sequence are significant differences between the Han and Tibetan populations, which is associated with low hemoglobin concentrations in the Tibetans [10]. In addition, the expression of integrins detected in RBCs are known to play a significant role in the adhesion of hematopoietic stem cells (HSCs). A overlapping integrin repertoire was observed in RBCs and stromal CD31^+^ HSCs [11], in which the *ITGA6* gene is important. This result indicates that *ITGA6* was associated with the production of erythropoiesis. Eto2 is a transcriptional corepressor involved in erythrocyte differentiation. Previous studies reported that *ERBB4* colocalized with Eto2 which regulated differentiation during erythropoiesis by repressing important genes [12]. Here, we conducted a study to investigate wether these genes associated with HAPC are variant in Chinese Han and Tibetan populations.

## RESULTS

The characteristics of HAPC patients and controls are presented in Table 1. The basic information of candidate SNPs in Han and Tibetan subjects are summarized in Table 2 and Table 3, respectively. The location information of candidate SNPs in the subjects are presented in Table 4. In Han populations, we found that the rs75591953 (OR = 0.474, 95% CI = 0.249-0.901, *p* = 0.021), rs75984373 (OR = 0.429, 95% CI = 0.229-0.804, *p* = 0.008) in *EPAS1*, rs6744873 (OR = 0.467, 95% CI = 0.226-0.964, *p* = 0.037) in *ITGA6*, rs17335043 (OR = 0.140, 95% CI = 0.036-0.548, *p* = 0.001) in *ERBB4* were significantly associated with decreased HAPC risk. Similarly, in Tibetan populations, the rs3749148 (OR = 0.522, 95% CI = 0.283-0.964, *p* = 0.037) in *ITGA6*, rs934607 (OR = 0.432, 95% CI = 0.227-0.823, *p* = 0.010), rs141267844 (OR = 0.439, 95% CI = 0.227-0.848, *p* = 0.013) in *ERBB4* were associated with decreased HAPC susceptibility. Moreover, the rs6710946 (OR = 5.182, 95% CI = 1.516-17.71, *p* = 0.004) in *ERBB4* was associated with increased HAPC risk.

**Table 1:** Basic characteristics of the control individuals and patients with high altitude polycythemia

Variables	Han		Tibetan	
	Case (n=70)	Control (n=30)	Case (n=70)	Control (n=30)
Sex				
Male	35	15	35	15
Female	35	15	35	15

**Table 2:** Basic information of candidate SNPs in Han subjects

SNP_ID	Gene	Alleles A/B	Case (N)			HWE Case	Control (N)			HWE Control	OR (95% CI)	*p* value
			AA	AB	BB		AA	AB	BB			
rs7571218	EPAS1	A/G	8	32	30	1.000	3	16	11	0.413	0.991(0.521-1.888)	0.979
rs6743991		A/C	1	11	58	0.458	1	6	23	1.000	0.887(0.320-2.460)	0.818
rs59901247		A/C	1	12	57	0.514	1	7	22	1.000	0.810(0.309-2.122)	0.667
rs7567582		T/C	5	23	42	0.506	3	9	18	0.596	1.068(0.514-2.216)	0.861
rs75591953		C/T	13	11	46	0.475	10	5	15	0.534	0.474(0.249-0.901)	**0.021**
rs79843796		C/G	6	4	60	0.000	5	5	20	0.040	0.498(0.213-1.167)	0.104
rs117227021		T/G	0	7	63	1.000	1	4	25	1.000	0.711(0.199-2.526)	0.596
rs35508970		C/T	0	11	59	1.000	1	5	24	1.000	0.904(0.299-2.727)	0.858
rs7557402		C/G	4	25	41	1.000	3	8	19	0.563	1.182(0.561-2.492)	0.660
rs75984373		C/T	16	10	44	0.656	12	5	13	0.356	0.429(0.229-0.804)	**0.008**
rs2272499	ITGA6	A/G	4	23	43	0.729	1	12	17	0.304	1.09(0.515-2.308)	0.821
rs55667609		A/G	1	8	61	0.292	1	2	27	1.000	2.154(0.457-10.15)	0.321
rs17676773		G/A	2	2	66	**0.002**	0	8	22	1.000	0.280(0.092-0.847)	0.018
rs1574028		C/A	0	3	67	1.000	0	3	27	1.000	0.613(0.010-3.769)	0.594
rs6716540		T/C	9	14	45	**0.001**	5	10	15	0.239	0.554(0.282-1.088)	0.084
rs3749148		T/G	21	28	21	0.098	5	18	6	0.278	1.071(0.581-1.977)	0.825
rs11895564		G/A	2	12	56	0.205	2	10	18	1.000	0.495(0.218-1.125)	0.089
rs3792259		G/C	0	11	59	1.000	1	4	25	1.000	1.151(0.351-3.775)	0.816
rs6744873		G/A	6	15	49	0.083	3	13	14	0.671	0.467(0.226-0.964)	**0.037**
rs145810451		A/G	1	1	68	**0.022**	1	6	23	1.000	0.190(0.046-0.787)	0.012
rs13002712	ERBB4	A/G	19	35	16	1.000	5	15	10	1.000	1.438(0.7764-2.664)	0.247
rs6735267		C/T	3	3	54	1.000	3	3	24	1.000	0.460(0.090-2.355)	0.340
rs35778743		T/C	0	1	69	1.000	1	1	28	1.000	0.410(0.025-6.669)	0.518
rs934607		A/G	8	26	36	0.392	1	13	16	0.287	1.484(0.726-3.033)	0.278
rs4672613		C/T	0	10	60	1.000	1	5	24	1.000	0.815(0.266-2.499)	0.721
rs17335043		A/C	4	3	63	1.000	2	8	20	1.000	0.140(0.036-0.548)	**0.001**
rs34621071		A/G	0	7	63	1.000	1	7	22	1.000	0.384(0.128-1.148)	0.077
rs141267844		A/T	6	27	37	0.767	1	17	12	0.064	0.931(0.474-1.830)	0.836
rs4673628		A/G	0	4	66	1.000	1	3	26	1.000	0.539(0.117-2.489)	0.422
rs6710946		T/C	8	21	41	0.065	2	13	15	0.636	1.030(0.513-2.069)	0.934

SNP: Single-nucleotide polymorphism; OR: odds ratio; 95% CI: 95% confidence interval; HWE: Hardy-Weinberg equilibrium; Site with HWE *p*≤0.05 excluded; *p*<0.05 indicates statistical significance for allele model.

**Table 3:** Basic information of candidate SNPs in Tibetan subjects

SNP_ID	Gene	Alleles A/B	Case (N)			HWE Case	Control (N)			HWE Control	OR (95% CI)	*p* value
			AA	AB	BB		AA	AB	BB			
rs7571218	EPAS1	G/A	2	27	41	0.493	1	7	22	0.505	1.612(0.715-3.634)	0.247
rs6743991		A/C	0	3	67	1.000	0	0	30	1.000	1.354(0.253-3.726)	0.253
rs59901247		A/C	2	7	61	**0.047**	0	4	26	1.000	1.194(0.364-3.911)	0.770
rs7567582		C/T	4	26	40	1.000	1	9	20	1.000	1.429(0.669-3.054)	0.356
rs75591953		T/C	3	8	59	**0.015**	0	2	28	1.000	3.222(0.709-14.64)	0.111
rs79843796		C/G	3	7	60	**0.001**	4	0	26	**0.000**	0.464(0.172-1.247)	0.121
rs117227021		T/G	1	1	68	**0.022**	0	1	29	1.000	1.292(0.132-12.68)	0.826
rs35508970		C/T	0	6	64	1.000	0	2	28	1.000	1.299(0.255-6.625)	0.753
rs7557402		G/C	7	26	37	0.556	1	11	18	1.000	1.446(0.707-2.957)	0.311
rs75984373		T/C	1	5	64	0.146	0	1	29	1.000	3.105(0.374-25.81)	0.270
rs2272499	ITGA6	A/G	2	14	54	0.302	2	9	19	0.589	0.533(0.242-1.174)	0.115
rs55667609		A/G	0	4	66	1.000	1	2	27	1.000	0.824(0.147-4.625)	0.825
rs17676773		G/A	0	13	57	1.000	0	6	24	1.000	0.921(0.333-2.551)	0.875
rs1574028		C/A	0	7	63	1.000	0	3	27	1.000	1.000(0.250-4.005)	1.000
rs6716540		T/C	6	30	34	0.318	5	15	10	1.000	0.571(0.289-1.128)	0.105
rs3749148		G/T	14	30	26	0.455	10	14	16	1.000	0.522(0.283-0.964)	**0.037**
rs11895564		G/A	1	17	52	1.000	0	11	19	0.551	0.700(0.310-1.578)	0.387
rs3792259		G/C	1	3	66	1.000	0	3	27	1.000	0.422(0.083-2.155)	0.286
rs6744873		G/A	3	27	40	0.072	3	14	13	1.000	0.651(0.320-1.321)	0.233
rs145810451		A/G	0	7	63	1.000	0	2	28	1.000	1.55(0.3124-7.687)	0.589
rs13002712	ERBB4	A/G	14	41	15	0.231	4	16	10	0.711	1.458(0.789-2.693)	0.228
rs6735267		C/T	8	13	49	1.000	3	3	24	1.000	1.464(0.638-2.573)	0.130
rs35778743		T/C	0	9	61	1.000	0	5	25	1.000	0.756(0.242-2.357)	0.629
rs934607		A/G	2	29	39	0.324	4	17	9	0.472	0.432(0.227-0.823)	**0.010**
rs4672613		C/T	1	10	59	0.402	0	4	26	1.000	1.312(0.406-4.247)	0.649
rs17335043		A/C	3	5	62	1.000	1	5	24	1.000	0.411(0.114-1.478)	0.162
rs34621071		A/G	0	8	62	1.000	0	2	28	1.000	1.758(0.362-8.533)	0.479
rs141267844		A/T	3	24	43	1.000	4	15	11	1.000	0.439(0.227-0.848)	**0.013**
rs4673628		A/G	0	2	68	1.000	0	3	27	1.000	0.275(0.045-1.692)	0.138
rs6710946		T/C	2	26	42	0.720	0	3	27	1.000	5.182(1.516-17.71)	**0.004**

SNP: Single-nucleotide polymorphism; OR: odds ratio; 95% CI: 95% confidence interval; HWE: Hardy-Weinberg equilibrium; Site with HWE *p*≤0.05 excluded; *p*<0.05 indicates statistical significance for allele model.

**Table 4:** Location information of candidate SNPs in this study

SNP_ID	Gene	Region	Position	MAF (Han)		MAF (Tibetan)	
				Case	Control	Case	Control
rs7571218	EPAS1	intronic	46605659	0.343	0.345	0.221	0.15
rs6743991		intronic	46583235	0.093	0.103	0.021	0.000
rs59901247		exonic	46609572	0.100	0.121	0.078	0.067
rs7567582		intronic	46602722	0.236	0.224	0.243	0.183
rs75591953		intronic	46583279	0.264	0.431	0.100	0.033
rs79843796		intronic	46609045	0.109	0.196	0.075	0.148
rs117227021		intronic	46605935	0.050	0.069	0.021	0.017
rs35508970		intronic	46583281	0.079	0.086	0.043	0.033
rs7557402		splicing	46603671	0.236	0.207	0.286	0.217
rs75984373		intronic	46583281	0.300	0.500	0.050	0.017
rs2272499	ITGA6	intronic	173332115	0.221	0.207	0.129	0.217
rs55667609		intronic	173341396	0.071	0.035	0.029	0.035
rs17676773		intronic	173333720	0.043	0.138	0.093	0.100
rs1574028		intronic	173333840	0.021	0.035	0.050	0.050
rs6716540		intronic	173292713	0.235	0.357	0.267	0.389
rs3749148		intronic	173330549	0.500	0.483	0.406	0.433
rs11895564		exonic	173339808	0.114	0.207	0.136	0.183
rs3792259		intronic	173362970	0.079	0.069	0.022	0.050
rs6744873		intronic	173292709	0.169	0.304	0.230	0.315
rs145810451		intronic	173338660	0.021	0.103	0.051	0.033
rs13002712	ERBB4	intronic	212587321	0.479	0.431	0.493	0.400
rs6735267		upstream	213403863	0.026	0.056	0.046	0.000
rs35778743		intronic	212589986	0.007	0.017	0.064	0.083
rs934607		intronic	212252809	0.300	0.224	0.236	0.417
rs4672613		intronic	212293044	0.071	0.086	0.086	0.067
rs17335043		intronic	212426466	0.023	0.143	0.037	0.086
rs34621071		intronic	212522651	0.050	0.121	0.057	0.033
rs141267844		intronic	212295590	0.279	0.293	0.214	0.383
rs4673628		intronic	212543924	0.029	0.052	0.014	0.050
rs6710946		intronic	212295875	0.264	0.259	0.214	0.050

MAF: Minor allele frequency

**Figure 1:**
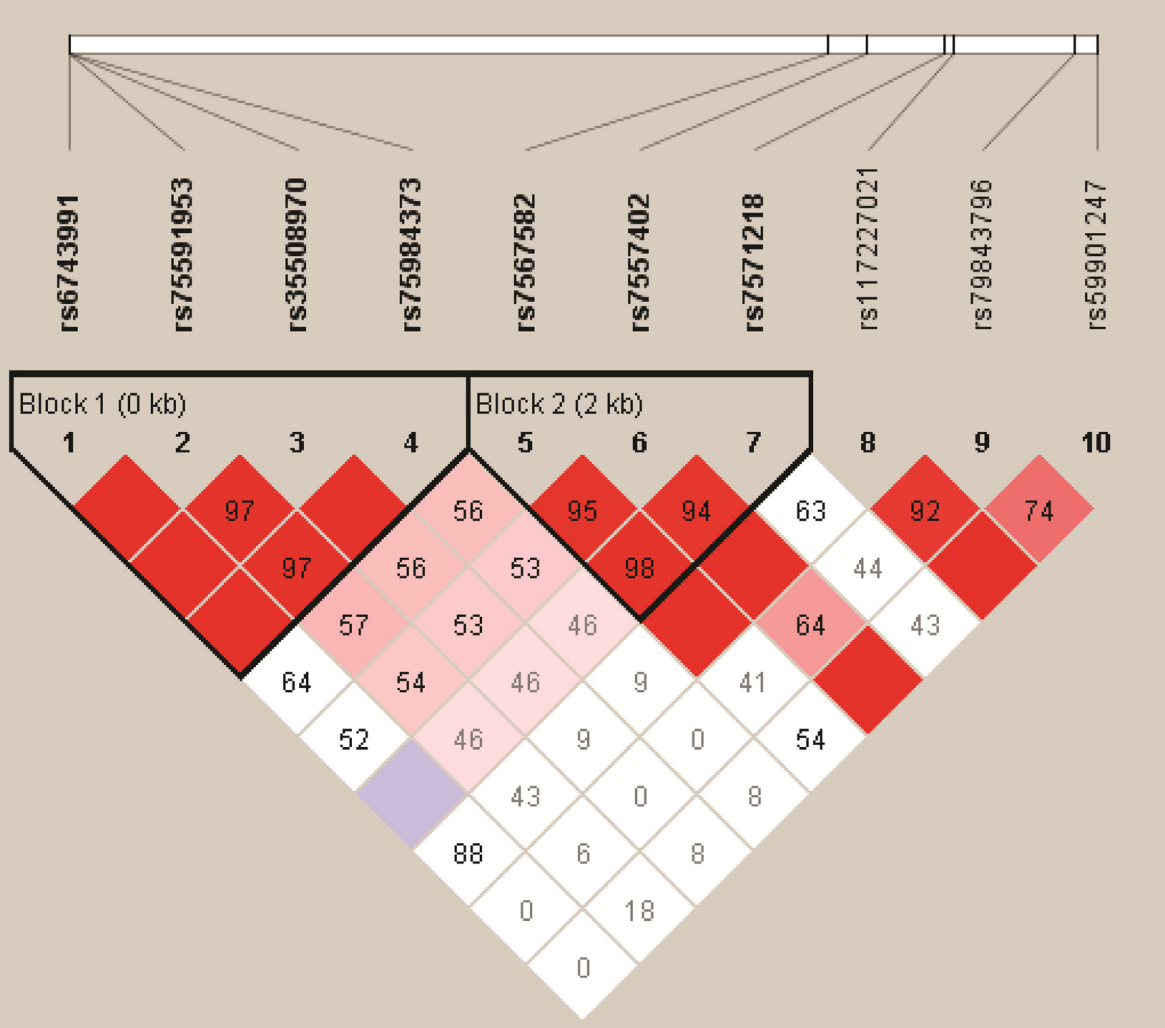
Haplotype block map for the ten *EPAS1* SNPs genotype in this study

**Figure 2:**
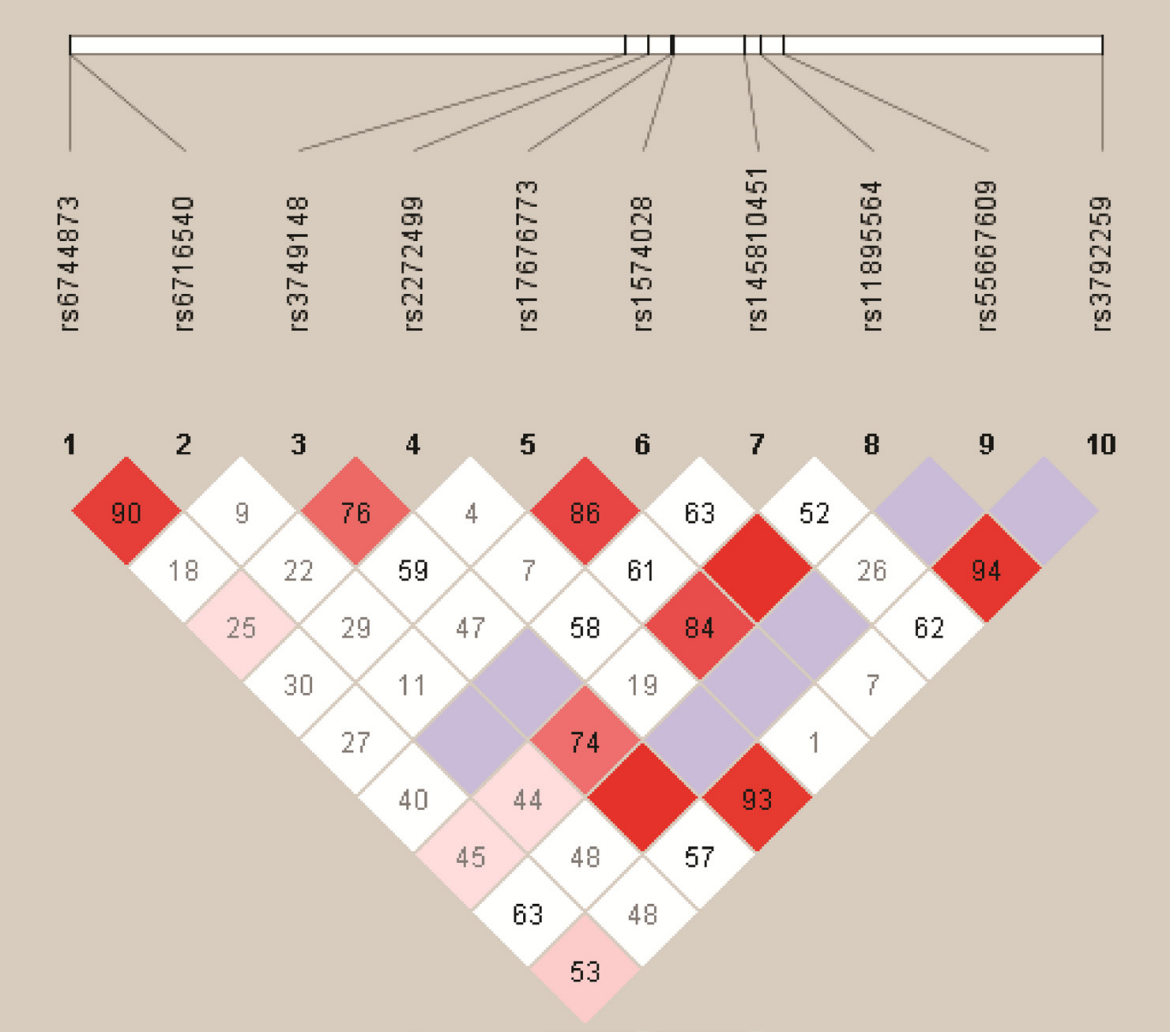
Haplotype block map for the ten *ITGA6* SNPs genotype in this study

**Figure 3:**
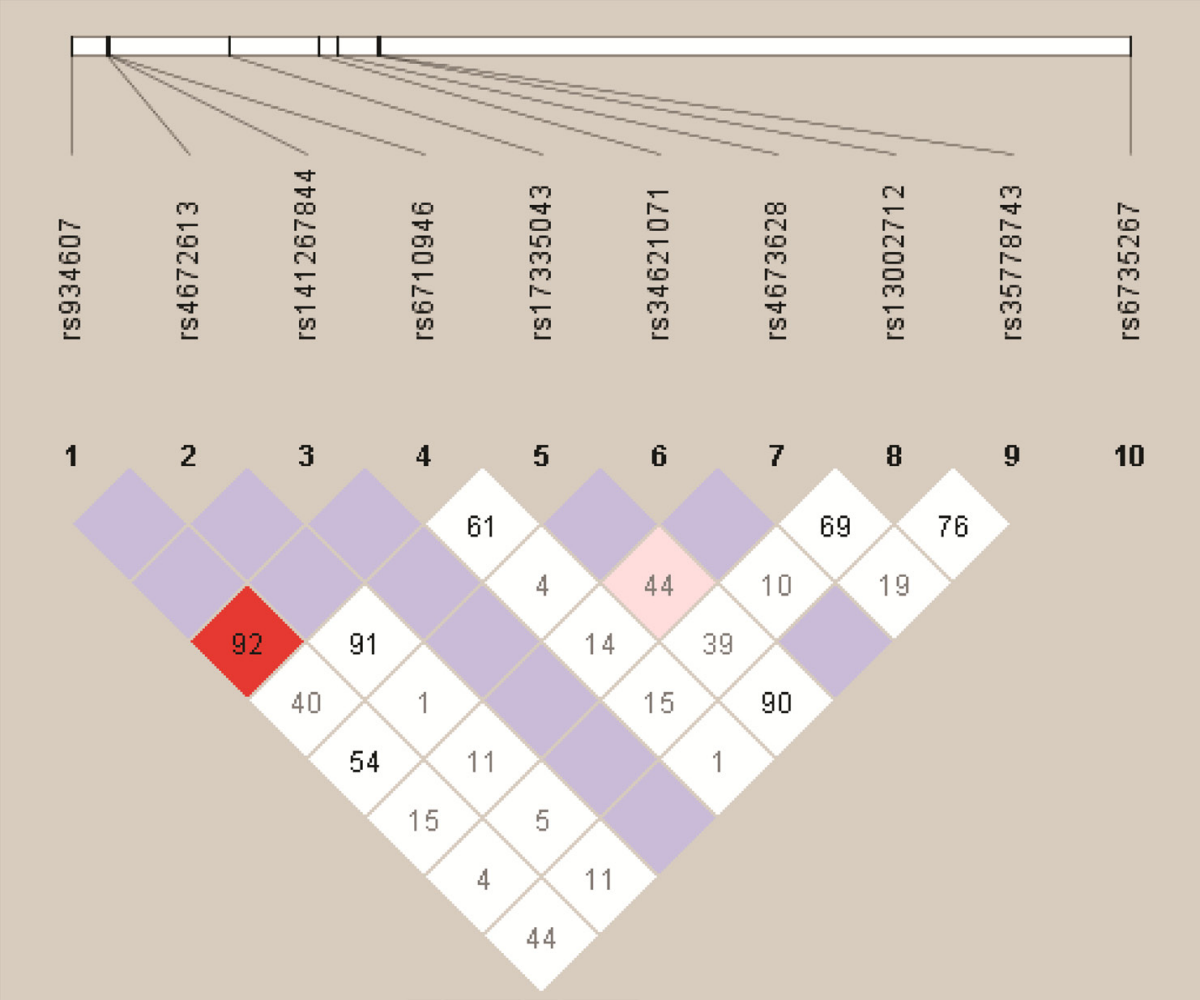
Haplotype block map for the ten *ERBB4* SNPs genotype in this study

We analyzed the association between SNPs and HAPC risk by unconditional logistic regression analysis using three models (dominant, recessive and additive model) in Han and Tibetan populations (Table 5 and Table 6). After stratifying by gender, in Han populations, we found the rs145810451 (*p* = 0.012, *p* = 0.042) in *ITGA6*, rs17335043 (*p* = 0.002, *p* = 0.007) in *ERBB4* were associated with a decreased risk of HAPC using the dominant and additive model, and the rs17676773 (*p* = 0.006) was associated with a reduced risk of HAPC in the dominant model. Moreover, in Tibetans, we found the rs934607 (*p* = 0.021, *p* = 0.008) and rs141267844 (*p* = 0.025, *p* = 0.016) in *ERBB4* were associated with a decreased risk of HAPC in the dominant and additive model. On the contrary, the rs6710946 (*p* = 0.006, *p* = 0.007) in *ERBB4* was associated with an increased risk of HAPC in the dominant and additive model. Furthermore, linkage disequiibrium (LD) analysis was done using genotype data from all the subjects. Two main linkage blocks were detected among the *EPAS1* SNPs (Figure 1). Block 1 contains rs6743991, rs75591953 and rs75984373, and block 2 contains rs7567582, rs7557402 and rs7571218. Another haplotype block that included twenty SNPs in *ITGA6* and *REBB4* are shown in Figure 2 and 3, respectively.

**Table 5:** Single loci associations with high altitude polycythemia risk in Han subjects

SNP_ID	Model	Ref Allele	Alt Allele	OR	95% CI	*p* value
rs17676773	Dominant	G	A	0.145	0.037-0.569	**0.006**
	Recessive			0.563	0.357-1.576	0.264
	Additive			0.343	0.112-1.047	0.060
rs145810451	Dominant	A	G	0.107	0.019-0.607	**0.012**
	Recessive			0.262	0.174-0.776	0.263
	Additive			0.229	0.055-0.949	**0.042**
rs17335043	Dominant	A	C	0.089	0.020-0.405	**0.002**
	Recessive			0.473	0.169-0.837	0.362
	Additive			0.248	0.034-0.525	**0.007**

OR: odds ratio; 95% CI: 95% confidence interval; p<0.05 indicates statistical significance for genetic model.

**Table 6:** Single loci associations with high altitude polycythemia risk in Tibetan subjects

SNP_ID	Model	Ref Allele	Alt Allele	OR	95% CI	*p* value
rs934607	Dominant	A	G	0.340	0.136-0.847	**0.021**
	Recessive			0.191	0.033-1.107	0.065
	Additive			0.364	0.172-0.770	**0.008**
rs141267844	Dominant	A	T	0.361	0.148-0.878	**0.025**
	Recessive			0.290	0.061-1.390	0.122
	Additive			0.425	0.212-0.853	**0.016**
rs6710946	Dominant	T	C	6.005	1.661-21.710	**0.006**
	Recessive			4.283	2.374-17.374	0.999
	Additive			5.752	1.621-20.410	**0.007**

OR: odds ratio; 95% CI: 95% confidence interval; *p*<0.05 indicates statistical significance for genetic model.

## DISCUSSION

In this study, we revealed associations between the polymorphisms of *EPAS1, ITGA6* and *ERBB4* and HAPC susceptibility, and we revealed several crucial findings. The SNPs examined (the rs75591953 and rs75984373 in *EPAS1*, the rs6744873 and rs3749148 in *ITGA6*, the rs17335043, rs934607, rs141267844 and rs6710946 in *ERBB4*) were strongly associated with HAPC. Taken together, these results suggested that polymorphisms in these three genes might play significant roles in HAPC in Chinese Han and Tibetan populations. It was reported that more than 12 million people live in the Qinghai-Tibet Plateau, most of people who settled here recent years are Chinese Han coming from low altitude areas. As known the higher altitude, the higher incidence of HAPC is, it is a disease that affects most individuals living at high altitudes. The majority of individuals can reach a high level of RBCs after long-term exposure to high altitude environment, because our body need more RBCs to carry oxygen under hypoxia conditions [13, 14]. However, the continued increase in the number of RBCs can result in serious complications such as the high level of testosterone [15], low sleep quality [16] and oxidative stress [17], all which are involve in the pathogenesis of HAPC, but the genetic basis of HAPC has not been studied extensively, especially in Han population. Hypobaric hypoxia is a major geographical feature in the plateau region [18]. In the plateau region, the long-term adaptation and natural selection of modern Tibetan and Han changed their genetic structure [19]. Chronic hypobaric hypoxia is the main reason of HAPC [20].

*EPAS1* is located at chromosome 2 and involved in RBC and Hb production. *EPAS1* is a very significant gene in the HIF pathway. HIF participate in the primary signaling pathway which is responsible for activating gene expression in response to oxygen levels. Gene-related studies have demonstrated that the non-coding nucleotide variants in *EPAS1* was associated with a reduced hemoglobin concentration in Tibetans [21, 22]. Moreover, the study suggested that *EPAS1* was a pivotal gene mutated in Tibetans, those who are blunted RBC response to hypoxemia. Previous study supported a role of *EPAS1* in maintaining what is believed to be a hallmark of altitude adaption in Tibetans: a blunted erythropoietic response to lower oxygen saturation values. By limiting hypoxia-induced RBC production, Himalayan highlanders avoid the rheological consequences of high hematocrit value to prevent excessive polycythemia [21]. Tibetans developed genes such as *EPAS1* might allow Tibetans to evolve more effective mechanisms and not to overproduce RBCs in response to altitude hypoxia, and to help them adapt to life in the thinner air [21, 22]. In addtion, erythrocytosis may be secondary to abnormal ventilation, which in turn stimulates the production of excess erythropoietin. Han populations in Tibet have lower ventilation and hypoxia ventilation response, resulting in excessive production of HAPC. In general, the Tibetan's hemoglobin concentration is about 1 g/dl which is lower than Andean populations at the same altitude. This shows that the Tibetans in the plateau hypoxic environment form a dull erythropoiesis reaction. Frank et al [23] reported the HIF has been implicated as the primary regulator of erythropoietin. They found the HIF2α, a subunit of HIF family, had a missense mutation so that compromised its hydroxylation, which is necessary to stable conformation and ability to induce erythrocytosis. In addition, the functional studies showed that wild-type HIF2α regulated the production of erythropoietin in adults. In summary, *EPAS1* gene have a significant influence for the production of erythrocytes.

The protein product of *ITGA6* is the integrin α6. Seagroves et al [24] reported that *ITGA6* is a direct transcription target for HIF transcription factors. Three putative hypoxia response elements were identified in the *ITGA6* promoter, two of which effectively bind HIF-lα or HIF-2α. As we all know, blood is one of the most intensely studies of human tissues, it has many functions in the body and consisits of erythrocytes. Red blood cells (also known as erythrocytes) are the most common type of blood cells and carry oxygen to the body tissues. HIF-1 and HIF-2 are transcription factors as the main regulator of hypoxia. Under normal oxygen pressure, von Hippel Lindau (VHL) protein binds to the HIF-α subunits and labels them by proteasome degradation. Proline hydroxylation of HIF-α by prolyl hydroxylases enzymes is required for the interaction of HIF-α with VHL protein [25]. In the above article, we have already mentioned that the HIF has been implicated in the primary regulation of erythropoietin and a missense mutation that compromised the hydroxylation of HIF2α, which allows both to maintain its stable conformation and its induction of erythrocytosis. Meanwhile, hematopoietic stem cells (HSCs) are rare cells in the bone marrow that are self-renewing and produce differentiated blood cells. During hematopoietic differentiation, the cells gradually expand the number and lose their pluripotency. Ultimately, HSCs can produce large amounts of bone marrow cells (such as erythrocytes). The expression of integrins, which are known to play a significant role in the adhesion of hematopoietic stem cells (HSCs), were detected in erythropoiesis [26]. *ITGA6* is a direct transcription target of HIF transcription factor, HIF-1 and HIF-2 are the main transcription factors in hypoxic environment, which are closely related to the formation of erythrocytes. To sum up, we have ample evidence to believe that in the hypoxic environment, that *ITGA6* gene was associated with the production of erythrocytes.

*ERBB* gene is an oncogene encoding human epidermal-growth factor receptor (HER), and different HER proteins have highly homologous amino acid sequences and similar structural features. *ERBB4* gene is one of the members. HER bind with ligand through the automatic phosphorylation and kinase cascade to transmit signals in the cell, ultimately regulate cell growth and division. Dudley et al [27] reported the proliferation of tumor endothelial cell was associated with overexpressed *ERBB4*. In contrast, normal endothelial cell are growth inhibited by neuregulin, whereas tumor endothelial cell are not affected. Higher levels of vascular endothelial growth factor receptors have been detected on tumor endothelial cell compared with normal endothelial cell. Therefore, *ERBB4* were strongly associated with vascular wall stability, we also believe that *ERBB4* expression was indirectly associated with production of erythrocytes. Previous studies reported that Eto2 is a transcriptional corepressor that is involved in erythrocyte differentiation. Bagheri et al [28, 29] demonstrated that variant of rs6735267 in *ERBB4* gene was associated with breast cancer and the variant of rs4673628 in *ERBB4* gene increases susceptibility to schizophrenia. In present, most of the reports on *ERBB4* gene are associated with breast cancer and schizophrenia. Based on the results of our research, we found that *ERBB4* gene polymorphism was associated with HAPC in Chinese Han and Tibetan populations.

Qinghai-Tibetan plateau is located at the southwest of China. It is the typical mountain plateau with biodiversity-rich, low temperature and hypoxia. Tibet is a mysterious land and the most biodiversity-rich place. Tibetans are the oldest indigenous mountainous population who settle down at least 500,000 years in Tibet. In this extreme hypoxic environment, the incidence of HAPC increased significantly. HAPC occurs among Tibetans at a lower incidence than Han Chinese migrants living in Tibet that may due to differences in geographical position and dietary habits. To further explore the associations of *EPAS1, ITGA6*, and *ERBB4* SNPs with HAPC in Chinese Han and Tibetan populations, larger samples and deeper mechanism researches are needed.

## MATERIALS AND METHODS

### Study population

For perform the study, the Chinese Han and Tibetan populations-based case–control study comprising HAPC patients from the Second People’s Hospital of Tibet Autonomous Region and Tibet military region general hospital. We recruited a total of 100 patients (70 Han, 30 Tibetan) with HAPC patients and 100 healthy control subjects (30 Han, 70 Tibetan), and all subjects were excluded from the study if they had an established diagnosis of chronic obstructive pulmonary disease, pulmonary infection, asthma, shunt conditions or congenital heart disease. Cases had not received any treatment before recruitment. There were no restrictions on recruitment in terms of age, gender, or clinical stage of disease. The aim is to reduce the therapeutic factors and potential environmental impacting the variation of HAPC. All Han subjects had emigrated from low altitude regions and lived at an altitude of more than 3600 meters for at least 3 months. HAPC patients were defined as having a hemoglobin concentration ≥210 g/L in males and ≥190 g/L in females. All the subjects reading and signing an informed consent form in this study, and the ethics Committee of Xizang Minzu University School of Medicine approved our use of blood samples and our protocol. All the participants are Chinese Han and Tibetan ethnic, and were informed the purpose and experimental procedures of the study.

### Epidemiological and clinical data

We collected demographic and clinical data using a standardized epidemiological questionnaire, including age, gender, race, place of residence, educational level, family cancer history and so on. We obtained clinical information for the patients through consulted with their treating physicians or from reviews of their medical charts, including blood oxygen saturation, hemoglobin and erythropoietin and so on. After signing an informed consent form, venous blood samples (5 ml) were obtained from each participant.

### SNP selection and genotyping

Thirty SNPs of three different genes were analyzed in this study. A total of ten SNPs in *EPAS1*, ten SNPs in *ITGA6* and ten SNPs in *ERBB4* with minor allele frequency (MAF) > 0.05 in the Asian population HapMap database. Genomic DNA was extracted from the peripheral blood of both the 100 HAPC patients and 100 healthy controls using the Gold Mag-Mini Purification Kit, and DNA concentrations were measured using the NanoDrop2000. Sequenom Mass ARRAY Assay Design3.0 software was used to design multiplexed SNP Mass EXTEND assay, and SNP genotyping was performed utilizing the Sequenom Mass ARRAY RS1000 recommended by the manufacturer.

### Statistical analysis

The SPSS 17.0 statistical software and Microsoft Excel were used for statistical analysis. The genotype frequencies of each SNP in the control subjects were checked using the Hardy–Weinberg equilibrium (HWE). We tested for differences in tSNP genotype distribution between patients and controls using the chi-square test. The effects of the polymorphisms on the risk of HAPC were expressed as odds ratios (ORs) with 95% confidence intervals (95% CIs), evaluated by three genetic models (dominant, recessive and additive model) using unconditional logistic regression analysis. We then stratified by sex and analyzed the association between genotype and HAPC risk using each of these three models. The Haploview software package and SHEsis software platform were used to assess LD analysis, haplotype construction, and the genetic association between polymorphisms. We used SNP Stats (Barcelona, Spain), a web-based software to test the associations between certain SNPs and the risk of HAPC in three genetic models (dominant, recessive, and additive). All p-values presented in this study were two sided, and we used p < 0.05 as the cut off value for statistical significance.

## CONCLUSION

In conclusion, our study suggest that a variation of *EPAS1, ITGA6*, and *ERBB4* may be involved in the genetic susceptibility to HAPC in Chinese Han and Tibetan populations. Further functional studies and larger population-based studies are required to further elucidate the biological pathways regulating HAPC susceptibility.
